# Prolonged In Vitro Exposure to Methylmalonic Acid Induces Inflammation, Glutamate Metabolism Disruption, and Alters Functional Gene Expression in C6 Astroglial Cells

**DOI:** 10.1007/s12640-026-00806-1

**Published:** 2026-06-23

**Authors:** Rômulo Rodrigo de Souza Almeida, Larissa Daniele Bobermin, Izaviany Schmitz, Filipe Renato Pereira Dias, Caio César Ramalho Bezerra, Mariana Rocke-Peters, Ester Rezena, Krista Minéia Wartchow, Fernanda Urruth Fontella, Diogo Onofre Souza, Moacir Wajner, Carlos-Alberto Gonçalves, Guilhian Leipnitz, André Quincozes-Santos

**Affiliations:** 1 Programa de Pós-Graduação em Ciências Biológicas: Bioquímica, Instituto de Ciências Básicas da Saúde, Universidade Federal do Rio Grande do Sul, Porto Alegre, RS Brazil; 2 Programa de Pós-Graduação em Neurociências, Instituto de Ciências Básicas da Saúde, Universidade Federal do Rio Grande do Sul, Porto Alegre, RS Brazil; 3 Departamento de Bioquímica, Laboratório de Neurotoxicidade e Glioproteção (LABGLIO), Instituto de Ciências Básicas da Saúde, Universidade Federal do Rio Grande do Sul, Rua Ramiro Barcelos, 2600 – Anexo, Bairro Santa Cecília, Porto Alegre, RS 90035–003 Brazil; 4 Programa de Pós-Graduação em Ciências Biológicas: Fisiologia, Instituto de Ciências Básicas da Saúde, Universidade Federal do Rio Grande do Sul, Porto Alegre, RS Brazil

**Keywords:** Methylmalonic acidemia, Astroglial cells, Neuroinflammation, Gliotoxicity

## Abstract

Methylmalonic acidemia is an inherited neurometabolic disorder characterized by accumulation of methylmalonic acid (MMA) in different tissues, particularly in the brain. As a result, patients frequently exhibit progressive neurological deterioration, accompanied by episodes of acute encephalopathy following metabolic decompensation. Astrocytes are glial cells that maintain the central nervous system homeostasis and may be important cellular targets of MMA-induced dysfunction. However, most in vitro experimental models for the study of methylmalonic acidemia are based on short-term exposure to the toxic metabolites that accumulate in patients. In this study, we used a prolonged experimental model, which has not been yet explored in the context of glial cells, focusing on the inflammatory response, glutamate metabolism, and putative signaling pathways that can contribute to understanding cellular damage observed in methylmalonic acidemia. It is emphasized that MMA is persistently elevated in the brain of the affected patients. Prolonged MMA exposure induced inflammation with significant increase in gene expression of cyclooxygenase 2, interleukin (IL)-1β and its receptor (IL1R1), and IL-6, accompanied by a decrease in IL-10 expression. MMA also increased glutamate uptake and the activity and gene expression of the enzyme glutamine synthetase, while it downregulated glial cell-derived neurotrophic factor (GDNF). The expression of NFκB, p38 MAPK, Nrf2, heme oxygenase 1, PGC-1α, and sirtuin 1 were also modulated by MMA treatment, indicating the critical role of these signaling pathways in the MMA-induced persistent gliotoxicity. Finally, it is conceivable that these changes may significantly contribute to clarify the pathogenesis of methylmalonic acidemia.

**Supplementary Information:**

The online version contains supplementary material available at 10.1007/s12640-026-00806-1.

## Introduction

Methylmalonic acidemia is an inherited metabolic disorder characterized by the accumulation of methylmalonic acid (MMA) in tissues and bodily fluids of patients, affecting approximately 1 in 80,000 individuals (Almási et al. 2019; Wajner 2019). This condition may arise from either a severe or complete deficiency of the activity of the enzyme methylmalonyl-CoA mutase (MUT, EC 5.4.99.2) or from defects in the synthesis of 5-deoxyadenosylcobalamin, a crucial cofactor for MUT (Manoli et al. 2016; Waisbren 2022). Symptoms typically present in the neonatal period, often following an episode of acute metabolic decompensation (Zwickler et al. 2012; Zhou et al. 2018). MMA is produced in tissues and subsequently distributed to the plasma and cerebrospinal fluid, where concentrations can rise to approximately 2.5–5.0 mmol/L during acute metabolic crises (Stabler et al. 1991; Hoffmann et al. 1993; Fontella et al. 2000; Wajner 2019). Importantly, due to the restricted efflux of dicarboxylates from the brain, MMA may accumulate to even higher concentrations in the central nervous system (Kölker et al. 2006; Sauer et al. 2010). Therefore, a prominent feature of methylmalonic acidemia is the neurological dysfunction, with patients frequently exhibiting manifestations such as cortical atrophy, lethargy, delayed psychomotor development, seizures, and coma, associated with progressive neurological deterioration and cortical atrophy (Yang et al. 2020; Chen et al. 2023).

Recent evidence describes the essential role of astrocytes in the neuropathology of methylmalonic acidemia (de Souza Almeida et al. 2022; Costa et al. 2023) These cells are the most versatile glial cell type in the central nervous system (CNS), providing structural, metabolic, trophic, and synaptic functions through the uptake of glutamate from the synaptic cleft and the release of gliotransmitters (Quincozes-Santos et al. 2021). Glutamate, the main excitatory neurotransmitter in the CNS, can trigger excitotoxicity when accumulated in the extracellular space, potentially contributing to the pathophysiology of methylmalonic acidemia (Costa et al. 2023; Magdaleno Roman and Chapa González 2024). Therefore, astroglial cells are critical in maintaining glutamate homeostasis by taking up extracellular glutamate and converting it into glutamine by the enzyme glutamine synthetase (GS, E.C. 6.3.1.2) (Quincozes-Santos et al. 2021, 2025). It is also noteworthy that, in response to brain injuries, astrocytes can become activated, fulfilling important functions in the inflammatory response and immunity by releasing a wide array of chemokines and cytokines (Colombo and Farina 2016; Bobermin et al. 2020).

Our group, recently, has demonstrated that MMA induces an inflammatory response and redox homeostasis disruption in C6 astroglial cells (de Souza Almeida et al. 2022). This cell line is widely utilized as an astrocyte-like model to investigate glial parameters and signaling pathways due to quickly response to cytotoxic agents and/or external stimuli, such as MMA (dos Santos et al. 2006; Quincozes-Santos et al. 2013; Bobermin et al. 2022a, b; Selistre et al. 2023). Noteworthy, most in vitro experimental models for the study of methylmalonic acidemia are based on short-term exposure of tissues or cells to toxins and/or metabolites that accumulate in patients, commonly lasting up to 24 h. However, in the present study, we extended the exposure period to 48 h, considering the 72-hour subculturing interval required for C6 astroglial cells. Thus, our study establishes a prolonged experimental model to characterize cellular damage in methylmalonic acidemia, which remains underexplored in the context of glial cells.

Therefore, the aim of the present study was to evaluate the changes in biomarkers of the inflammatory response and its potential molecular regulatory mechanisms, including interleukin-1β (IL-1β), interleukin-6 (IL-6), interleukin-10 (IL-10), tumor necrosis factor α (TNF-α), cyclooxygenase 2 (COX-2), nuclear factor kappa B (NFκB), nuclear factor erythroid 2-related factor 2 (Nrf2), heme oxygenase 1 (HO-1), p38 mitogen-activated protein kinases (p38 MAPK), peroxisome proliferator-activated receptor gamma coactivator 1-alpha (PGC-1α) and sirtuin 1 (SIRT1). Additionally, we assessed redox homeostasis and glutamatergic parameters, such as glutamate uptake, mRNA expression of excitatory amino acid carrier 1 (EAAC1), activity and mRNA expression of GS, as well as extracellular levels of glutamate and glutamine. Finally, we evaluated the expression of glial cell-derived neurotrophic factor (GDNF), a key molecule of neural functionality.

## Materials and Methods

### C6 Astroglial Cell Culture and Treatment

C6 astroglial cells were maintained following protocols established in our previous publications (dos Santos et al. 2006; Bobermin et al. 2022a, b; Dias et al. 2022). Briefly, the cells were seeded at a density of 5 × 10³ cells/cm² in 24- or 6-well plates (Corning/Falcon, Glendale, AZ, USA) and cultured for approximately three days in DMEM (Gibco catalog number 31600034, Waltham, MA, USA), supplemented with 5% fetal bovine serum (FBS, Qualified; Gibco catalog number 12657029, Waltham, MA, USA), at 37 °C in a 5% ­ CO_2_ incubator. Once the cells reached confluence, the culture medium was replaced with serum-free DMEM, and the cells were incubated for 48 h in the absence or presence of 5 mM MMA (Sigma-Aldrich catalog number M54058, Saint Louis, MO, USA). Each C6 cell culture plate represented one independent biological replicate (n), and all treatments were carried out in duplicate.

### Astrocyte Primary Cultures

Newborn (1–2 days old) Wistar rats had their cortices aseptically dissected from cerebral hemispheres, followed by meninges removal. The tissues were digested in Hank’s balanced salt solution (HBSS) containing 0.003% DNase using trypsin (0.05%) as previously described (Bobermin et al. 2020). After mechanical dissociation and centrifugation, the cells were resuspended in DMEM/F12 (Gibco) [10% fetal bovine serum (FBS), 15 mM HEPES, 14.3 mM ­ NaHCO_3_, 2.5 µg/mL amphotericin B, and 0.05 mg/mL gentamicin], plated on 6- or 24-well plates pre-coated with poly-L-lysine at a density of 5 × 10^5^ cells/cm^2^. Astrocytes were cultured at 37 °C in a 5% ­ CO_2_ incubator. After 24 h, the culture medium was exchanged; during the first week, the medium was replaced once every 2 days, and from the second week on, once every 4 days. From the third week on, astrocytes reached confluence, when were used for the experiments. The culture medium was replaced with serum-free DMEM, and the cells were incubated for 48 h in the absence or presence of 5 mM MMA (Sigma-Aldrich catalog number M54058, Saint Louis, MO, USA). Each cell culture plate represented one independent biological replicate (n), and all treatments were carried out in duplicate.

### MTT Reduction Assay

For the MTT reduction assay, C6 astroglial cells and primary astrocyte cultures were seeded at identical densities across all experimental conditions in 24-well plates (5 × 10³ cells/cm² and 5 × 10⁵ cells/cm², respectively). MTT (methylthiazolyldiphenyl-tetrazolium bromide, Sigma-Aldrich) was added to the culture medium at a concentration of 50 µg/mL and cells were incubated for 30 min at 37 °C in an atmosphere of 5% ­ CO_2_. Subsequently, the medium was removed, and the MTT crystals were dissolved in dimethyl-sulfoxide. Absorbance values were measured at 560 nm and 650 nm (Longoni et al. 2018). The results are expressed as percentages relative to the control conditions.

### DCFH Oxidation

For the DCFH oxidation assay, C6 astroglial cells were seeded at identical densities across all experimental conditions in 24-well plates (5 × 10³ cells/cm²). Intracellular reactive oxygen species (ROS) levels were detected using DCFH-DA (Sigma-Aldrich). DCFH-DA was added to the medium at a concentration of 10 µM after MMA exposure, and C6 astroglial cells were incubated for 30 min at 37 °C. Following DCFH-DA exposure, the cells were scraped into phosphate-buffered saline with 0.2% Triton X-100. The fluorescence was measured with excitation at 485 nm and emission at 520 nm (Bobermin et al. 2022a, b). Fluorescence values were normalized to the total protein content of each sample, and the resulting values were expressed as percentages relative to the control condition.

### JC‑1 Assay

For the JC-1 oxidation assay, C6 astroglial cells were seeded at identical densities across all experimental conditions in 24-well plates (5 × 10³ cells/cm²). To determine the mitochondrial membrane potential (ΔΨm), cells were incubated for 30 min with JC-1 (Invitrogen, 2 µg/ mL) after MMA exposure (Bobermin et al. 2022a, b). Then, cells were washed once with Hank’s balanced salt solution (HBSS) and the fluorescence was immediately read using an excitation wavelength of 485 nm and emission wavelengths of 540 and 590 nm. The ΔΨm was calculated using the ratio of 595 nm (red fluorescent J-aggregates) to 535 nm (green monomers). The resulting values were expressed as percentages relative to the control condition.

### Inflammatory Response Measurement

For extracellular cytokine level measurement, C6 astroglial cells were seeded at identical densities across all experimental conditions in 24-well plates (5 × 10³ cells/cm²). After MMA exposure, the extracellular medium was collected and stored at − 80 °C until analysis. For ELISA assays, 100 µL of each sample was used, and all samples were assayed in duplicate. ELISA kits for tumor necrosis factor-α (TNF-α; catalog #88-7340-22) and interleukin-1β (IL-1β; catalog #BMS630) were purchased from Invitrogen (Waltham, MA, USA). TNF-α and IL-1β concentrations were determined using separate standard curves generated for each cytokine in parallel with the samples. Results are presented as pg/mL.

### RNA Extraction and Quantitative RT-PCR

For gene expression analysis, total RNA was isolated from C6 astroglial cells (seeded in 6-well plates at a density of 5 × 10³ cells/cm²) and primary astrocytes (seeded in 6-well plates at a density of 5 × 10⁵ cells/cm²) using 1 ml of TRIzol reagent (Invitrogen, Waltham, MA, USA) following the manufacturer’s protocol, with chloroform extraction and isopropanol precipitation. The concentration and purity of the RNA were determined spectrophotometrically at ratio of 260:280 (all samples exhibited an A260/A280 ratio ≥ 1.9). Then, 2 µg of total RNA from each sample, contained in a volume of 10 µL, was reverse-transcribed using the Applied Biosystems High-Capacity Complementary DNA (cDNA) Reverse Transcription Kit (Applied Biosystems catalog number catalog number 4368814, Foster City, CA, USA) in a 20 µL reaction according to the manufacturer´s instructions. The messenger RNA (mRNA) encoding the target genes was quantified using the TaqMan real-time RT-PCR system with inventory primers and probes listed in Table 1, purchased from Applied Biosystems (Foster City, CA, USA). Quantitative RT-PCR was performed using 1:20 diluted cDNA samples, in duplicate, on the Applied Biosystems StepOnePlus Real-Time PCR System (Foster City, CA, USA). The upper limit for Ct value acceptance was set at 35, and mean Ct values for each gene are also reported in Table 1. Target mRNA levels were normalized to β-actin levels and expressed relative to control conditions using the 2^−ΔΔCt^ method (Livak and Schmittgen 2001).

**Table 1:** Genes analyzed by quantitative RT-PCR

mRNA target	TaqMan assay ID	Mean Ct values
β-actin	Rn00667869_m1	16.13*
Cyclooxygenase 2 (COX-2)	Rn01483828_m1	32.88
Excitatory amino acid carrier 1 (EAAC1)	Rn00564705_m1	22.42
Glutamine synthetase (GS)	Rn01483107_m1	20.94
Glial cell line-derived neurotrophic factor (GDNF)	Rn07311775_m1	30.95
Heme oxygenase (HO-1)	Rn01536933_m1	25.43
Interleukin-1β (IL-1β)	Rn00580432_m1	33.51
Interleukin-6 (IL-6)	Rn01410330_m1	30.65
Interleukin-10 (IL-10)	Rn00563409_m1	34.90
Interleukin 1 receptor type 1 (IL1R1)	Rn00565482_m1	33.29
Inducible nitric oxide synthase (iNOS)	Rn00561646_m1	26.77
Nuclear factor erythroid 2-related factor 2	Rn00582415_m1	21.87
Nuclear factor-kappa B (NFκB p65)	Rn01502266_m1	20.79
NADPH oxidase 2 (NOX-2)	Rn00576710_m1	32.87
p38 mitogen-activated protein kinases (p38 MAPK)	Rn00578842_m1	31.78
Peroxisome proliferator-activated receptor gamma coactivator 1- α (PGC-1α)	Rn00580241_m1	34.54
Sirtuin 1 (SIRT1)	Rn01428096_m1	26.13
Tumor necrosis factor α (TNFα)	Rn99999017_m1	34.12
Tumor necrosis factor receptor 1 (TNFR1)	Rn01492348_m1	31.63

* The mean Ct value was 16.11 for the control group and 16.15 for the MMA group

### Western Blotting

For Western blot analysis, C6 astroglial cells were seeded in 6-well plates at a density of 5 × 10³ cells/cm². The protocol was performed as previously described (Gayger-Dias et al. 2024). After treatment, cells were lysed in 100 µL of lysis buffer containing 4% SDS, and protein extracts were denatured for 5 min at ≥ 95 °C. Subsequently, the samples were centrifuged at 10,000×g for 5 min (Eppendorf, Centrifuge 5417R, Hamburg, Germany) and the supernatant was aliquoted, of which 5 µL were used for protein dosage. Equal amounts of total protein (10 µg per sample) were subjected to SDS-polyacrylamide gel electrophoresis and transferred to 0.45 μm pore-size nitrocellulose membranes. The transfer was confirmed using Ponceau S and the membranes were blocked for 2 h at room temperature with a 2% bovine serum albumin solution in tris-buffered saline containing Tween-20 (TBS-T; 10 mM Tris, 150 mM NaCl, 0.05% Tween-20, pH 7.5). The membranes were incubated overnight (4 °C) with anti-NFκB (dilution 1:1000; Cell Signaling Technology, catalog number 436700), anti-p38 MAPK (dilution 1:500; Cell Signaling Technology, catalog number 8690 S), and anti-PGC-1α (dilution 1:1000; ST Biotechnology, catalog number ST1202). Subsequently, the membranes were incubated with horseradish peroxidase-conjugated secondary antibodies (dilution 1:10,000; anti-mouse IgG, Elabscience, catalog number E-AB-1001; or anti-rabbit IgG, Elabscience, catalog number E-AB-1058) for 2 h at room temperature under shaking. For signal detection, the ECL Super Signal West Pico Plus (Thermo Fisher, catalog number 34580) was used and detected by Chemidoc MP (Bio-Rad Laboratories). The nitrocellulose membranes were reused after hydrogen peroxide stripping (35%; 10 min at 37 °C) for the different primary antibodies, and were washed successively with TBS-T. The membranes were then reprobed with mouse anti-β-actin antibody (Santa Cruz Biotechnology, catalog number sc-4778; dilution 1:10000) or 2 h at room temperature under shaking. Subsequently, the membranes were incubated with the appropriate horseradish peroxidase-conjugated secondary antibody (dilution 1:10,000; Elabscience, catalog number E-AB-1058) for 2 h at room temperature under shaking. The analysis was performed using ImageJ software with data normalization by β-actin content. Complete immunoblots corresponding to all quantitative data from this study are compiled in Supplementary Fig. 3 (Fig. S3).

### p65 NFκB Nuclear Content

Nuclear content was obtained from a 150 µL aliquot of astroglial cells and primary astrocytes seeded at identical densities across all experimental conditions in 6-well plates (5 × 10³ cells/cm² and 5 × 10⁵ cells/cm², respectively). Cells were lysed in PBS and treated with 1% Igepal CA-630 (1:1, v/v), followed by vortexing for 30 s and centrifugation at 400 × g for 5 min (Eppendorf, Centrifuge 5417R, Hamburg, Germany). After centrifugation, nuclei pellets were resuspended in specific solution (provided by ELISA kit manufacturer) and assayed for p65 NFκB using a commercial ELISA kit with some modifications (Elabscience, catalog #E-EL-R0674). Sample concentrations were determined using a standard curve, and the calculated values were normalized and expressed as a percentage of the control group.

### Nrf2 Nuclear Content

Nuclear content was obtained from a 150 µL aliquot of astroglial cells and primary astrocytes seeded at identical densities across all experimental conditions in 6-well plates (5 × 10³ cells/cm² and 5 × 10⁵ cells/cm², respectively). Cells were lysed in PBS and treated with 1% Igepal CA-630 (1:1, v/v), followed by vortexing for 30 s and centrifugation at 400 × g for 5 min (Eppendorf, Centrifuge 5417R, Hamburg, Germany). After centrifugation, nuclei pellets were resuspended in specific solution (provided by ELISA kit manufacturer) and assayed for Nrf2 using a commercial ELISA kit with some modifications (Bio-Techne-Novis Biologicals, catalog #NBP3-08161). Sample concentrations were determined using a standard curve, and the calculated values were normalized and expressed as a percentage of the control group.

### Glutamate Uptake Assay

C6 astroglial cells, seeded at identical densities across all experimental conditions in 24-well plates (5 × 10³ cells/cm²), were incubated at 37 °C in HBSS. The assay was performed in duplicate and initiated by the addition of L-glutamate (final concentration: 0.1 mM) and L-[3,4-³H] glutamate (final concentration: 0.33 µCi/mL; PerkinElmer catalog number NET490005MC, Boston, MA, USA) to a final volume of 0.3 mL per well. The assay was terminated after 10 min by removing the medium and rinsing the cells three times with ice-cold HBSS (0.3 mL per well). The cells were then lysed in 0.3 mL of a solution containing 0.5 M NaOH. Glutamate uptake was calculated by subtracting the non-specific uptake, determined using a solution with N-methyl-D-glucamine (dos Santos et al. 2006). Radioactivity was measured using a scintillation counter (PerkinElmer, Boston, MA, USA), and results are expressed as nmol/mg protein/minute.

### Glutamine Synthetase (GS) Activity

Cells cultured in 24-well plates at a density of 5 × 10³ cells/cm² and treated in duplicate were homogenized in 0.2 mL of 50 mM imidazole buffer (50 mM). An aliquot of 100 µL of this homogenate was then incubated with a reaction solution containing (mM): 50 imidazole, 50 hydroxylamine, 100 L-glutamine, 25 sodium arsenate dibasic heptahydrate, 0.2 ADP, and 2 manganese chloride, adjusted to pH 6.2, in a final reaction volume of 0.2 mL (Minet et al. 1997). After a 15-minute incubation at 37 °C, the reaction was terminated by adding 200 µL of a mixture containing 0.37 M ferric chloride, 0.67 M HCl, and 200 mM trichloroacetic acid. Following centrifugation at 1,000 × g for 15 min (Eppendorf, Centrifuge 5417R, Hamburg, Germany), the absorbance of the supernatant was measured at 540 nm (SpectraMax i3, Molecular Devices, San Jose, CA, USA) and compared to absorbance values obtained from standard quantities of γ–glutamyl hydroxamate (0.156–10 mM) added to the ferric chloride reagent. The activity is expressed as µmol/mg protein/h.

### Measurement of Extracellular Levels of Amino Acids

The concentrations of the free amino acids glutamate and glutamine were determined in the extracellular culture medium, collected from C6 astroglial cells seeded at identical densities across all experimental conditions in 24-well plates (5 × 10³ cells/cm²), were determined using high-performance liquid chromatography (HPLC) (Santos et al. 2018). Samples were derivatized with ο-phthalaldehyde (OPA), and separation was carried out with a reverse phase column (Supelcosil LC-18, 5 μm, 250 mm×4.6 mm, Supelco; Sigma-Aldrich; Merck KGaA, Darmstadt, Germany) in a Class VP Shimadzu Instruments liquid chromatograph with auto-injection (SIL-10AF, 50 µL loop valve injection, 40 µL injection volume), and fluorescence detection after pre-column derivatization with 100.5 µL OPA (5.4 mg OPA in 1 mL 0.2 M sodium borate pH 9.5) plus 25.5 µL 4% mercaptoethanol for 3 min. The mobile phase flowed at a rate of 1.4 mL/minute, and the column temperature was 24 °C (column oven, CTO-20AC). The buffer compositions were as follows: A: 0.04 mol/L sodium dihydrogen phosphate monohydrate buffer, pH 5.5, containing 80% methanol; B: 0.01 mol/L sodium dihydrogen phosphate monohydrate buffer, pH 5.5, containing 20% methanol. The gradient profile was modified according to the content of buffer B in the mobile phase: 100% at 0.1 min, 90% at 15 min, 10% at 48 min, 100% at 55–60 min. Absorbance was read at an excitation of 360 nm and an emission of 455 nm in a Shimadzu fluorescence detector (RF-10AxL Shimadzu Corporation, Kyoto, Japan). Samples of 20 µL were used in a 1:100 dilution and assayed in triplicate. Amino acids were identified by their retention time and were quantitatively determined by using their chromatographic peak area. A known amino acid standard mixture was used for calibration, and concentrations are expressed in µM.

### Protein Content

The protein content was determined using the Lowry method, with bovine serum albumin (BSA) fraction V (Inlab, São Paulo, SP, Brazil) as the standard (Lowry et al. 1951). For the assay, 20 µL of each sample was used, and all samples were assayed in duplicate.

### Statistical Analysis

Results are presented as mean ± standard deviation (S.D.). To ensure adequate power for statistical analysis at least five independent experiments were performed. For each experiment, replicates are noted in the figure legends. The normal distribution was confirmed by the Shapiro-Wilk test and variance homogeneity was tested using Bartlett’s test. Differences between control and MMA were statistically analyzed using Student’s t test for independent samples, except for cellular viability, which was performed by one-way ANOVA followed by Tukey’s test. All analyses were performed using the GraphPad Prism software version 9 (GraphPad Software, Inc., La Jolla, CA, USA). The p-values, t-values, and degrees of freedom are reported in the Results section. In the figures, *p* < 0.05 (*), *p* < 0.01 (**), *p* < 0.001 (***), and *p* < 0.0001 (****) indicate statistically significant differences from the control.

## Results

### MMA Exposure Increased Inflammatory Response in C6 Astroglial Cells and in Primary Astrocyte Cultures

**Fig. 1:**
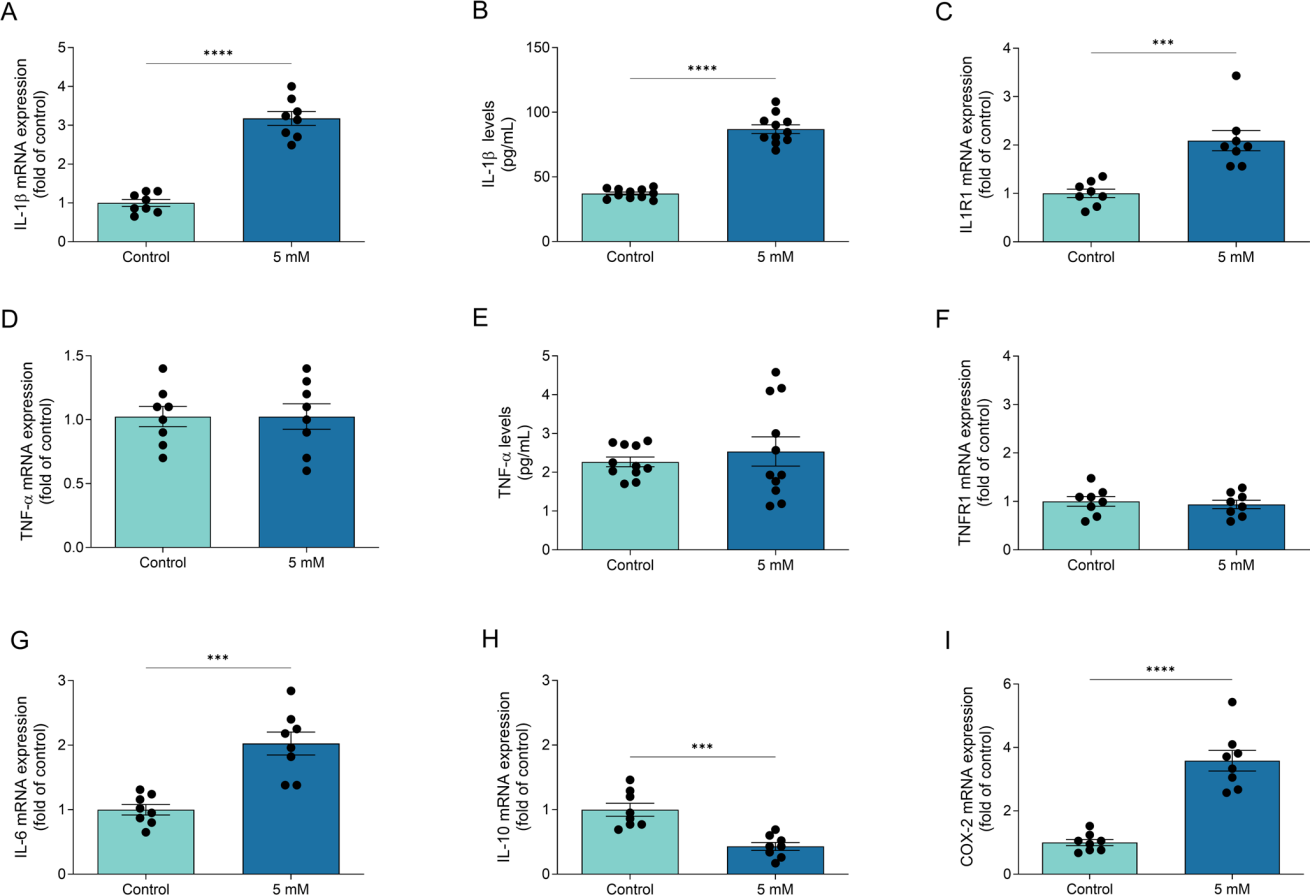
MMA treatment increased neuroinflammatory response. C6 astroglial cells were exposed to 5 mM MMA for 48 h and mRNA expression and extracellular levels of cytokine were measured. **A** mRNA expression of IL-1β, (**B**) extracellular levels of IL-1β, (**C**) mRNA expression of IL1R1, (**D**) mRNA expression of TNF-α, (**E**) extracellular levels of TNF-α, (**F**) mRNA expression of TNFR1, (**G**) mRNA expression of IL-6, (**H**) mRNA expression of IL-10, (**I**) mRNA expression of COX-2. Results are presented as mean ± standard deviation (S.D.) of at least seven independent experiments performed in duplicate. Differences between groups were statistically analyzed using Student’s t-test for independent samples. Values of *p* < 0.05 were considered statistically significant (* p < 0.05, ** p < 0.01, *** p < 0.001, and **** p < 0.0001)

Our prolonged in vitro experimental model shows that MMA exposure significantly increased both mRNA expression (t(14) = 10.88, *p* < 0.0001, Fig. 1A) and extracellular levels (t(20) = 13.96, *p* < 0.0001, Fig. 1B) of the classical pro-inflammatory cytokine IL-1β, as well as the mRNA expression of IL1R1 [t(14) = 4.785, *p* = 0.0003; Fig. 1C], a member of the interleukin-1 receptor family. Interestingly, MMA did not change the mRNA expression [t(14) = 0.000, *p* > 0.9999; Fig. 1D] and levels [t(20) = 0.6711, *p* > 0.05; Fig. 1E] of TNF-α, as well as the expression of its receptor, TNFR1 [t(14) = 0.4744, *p* > 0.05; Fig. 1F]. In addition, MMA induced the mRNA expression of IL-6 [t(14) = 5.275, *p* = 0.0001; Fig. 1G], an interleukin rapidly produced in response to injury, while downregulated the mRNA of IL-10 [t(14) = 4.854, *p* = 0.0003; Fig. 1H], an anti-inflammatory cytokine. MMA also upregulated the inflammatory enzyme COX-2 [(t(14) = 7.576, *p* < 0.0001; Fig. 1I). It is important to note that 5 mM of MMA, as well as other concentrations such as 1 mM and 10 mM, did not alter the cellular viability (Fig. S1), in accordance with our previous publication (de Souza Almeida et al. 2022). Although 5 mM is a high concentration, it can be achieved during a metabolic crisis.

In order to validate the key findings of MMA in glial cells, we further assessed the expression of inflammatory mediators in primary astrocyte cultures (Fig. S2). Initially, cell viability was determined by MTT assay following 48 h exposure to increasing concentrations of MMA (1, 5, and 10 mM). Primary astrocytes exhibited a response profile comparable to that observed in C6 astroglial cells (Fig. S1); therefore, 5 mM MMA was selected for subsequent experiments. MMA increased the mRNA expression of pro-inflammatory mediators, including IL-1β [t(8) = 4.677, *p* = 0.0016; Fig. S2A] and IL-6 [t(8) = 10.47, *p* < 0.0001; Fig. S2B], while TNF-α expression remained unchanged [t(8) = 1.688, *p* = 0.1298; Fig. S2C].

### Molecular Mechanisms Involved in the Inflammatory Response and Redox Homeostasis

**Fig. 2:**
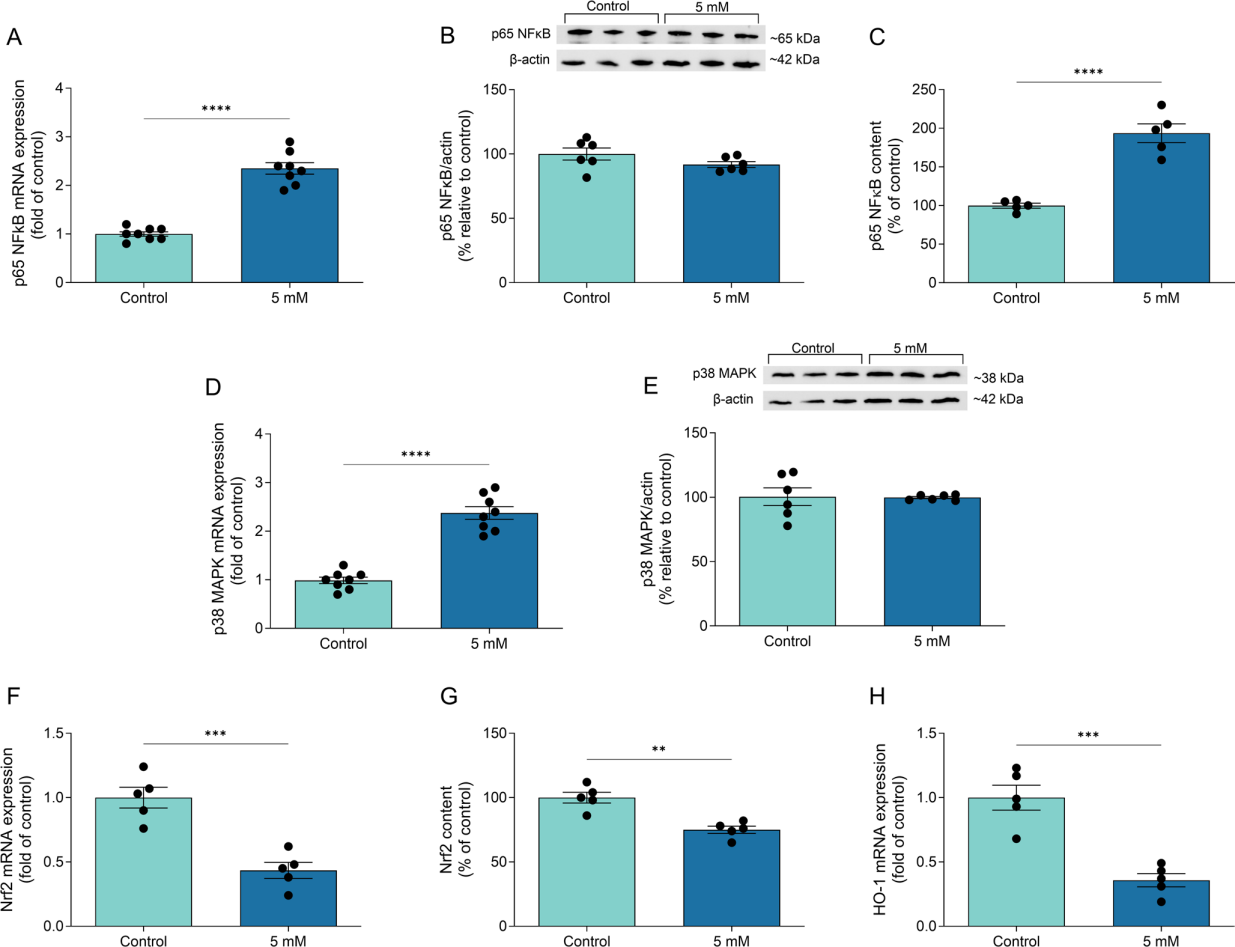
Signaling mechanisms involved in MMA-induced neuroinflammation. C6 astroglial cells were exposed to 5 mM MMA for 48 h and the mRNA expression of p65 NFκB (**A**), p38 MAPK (**D**), Nrf2 (**F**), and HO-1 (**H**); the protein levels of p65 NFκB (**B**), and p38 MAPK (**E**); the nuclear content of p65 NFκB (**C**) and Nrf2 (**G**) were measured. Results are presented as mean ± standard deviation (S.D.) of at least five independent experiments performed in duplicate. Differences between groups were statistically analyzed using Student’s t-test for independent samples. Values of *p* < 0.05 were considered statistically significant (** p < 0.01, *** p < 0.001, and **** p < 0.0001)

To characterize the molecular mechanisms involved in the effects of MMA, we assessed parameters related to the NFκB pathway. MMA significantly increased NFκB p65 mRNA levels [t(14) = 10.65, *p* < 0.0001; Fig. 2A], a key transcription factor that controls inflammatory gene expression. Despite no detectable change in total NFκB p65 protein levels by western blot following MMA exposure (Fig. 2B), nuclear NFκB p65 content, assessed by ELISA, was significantly elevated [t(8) = 7.439, *p* < 0.0001; Fig. 2C]. Of note, these findings were confirmed in primary astrocyte cultures (Fig. S2), where MMA also increased NFκB p65 mRNA levels [t(8) = 7.995, *p* < 0.0001; Fig. S2D], accompanied by a corresponding rise in its nuclear content [t(8) = 6.808, *p* = 0.0001; Fig. S2E].

p38 MAPK, a key mitogen-activated protein kinase involved in inflammation, exhibited increased gene expression following MMA exposure [t(14) = 9.464, *p* < 0.0001; Fig. 2D], whereas its total protein content remained unchanged (Fig. 2E). The transcription factor Nrf2 and its downstream effector HO-1 play a central role in regulating NFκB transcriptional activity, inflammatory responses, and mitochondrial function. In this context, MMA significantly decreased Nrf2 mRNA levels [t(8) = 5.543, *p* = 0.0005; Fig. 2F] and its nuclear content [t(8) = 4.903, *p* = 0.0012; Fig. 2G]. Consistently, HO-1 mRNA expression was also markedly downregulated [t(8) = 5.831, *p* = 0.0004; Fig. 2H]. These findings were also confirmed in primary astrocyte cultures (Fig. S2), where both Nrf2 mRNA expression [t(8) = 5.889, *p* = 0.0004; Fig. S2F] and nuclear content [t(8) = 5.462, *p* = 0.0006; Fig. S2G] were significantly reduced. Similarly, the expression of the cytoprotective enzyme HO-1 was decreased [t(8) = 5.343, *p* = 0.0007; Fig. S2H].

**Fig. 3:**
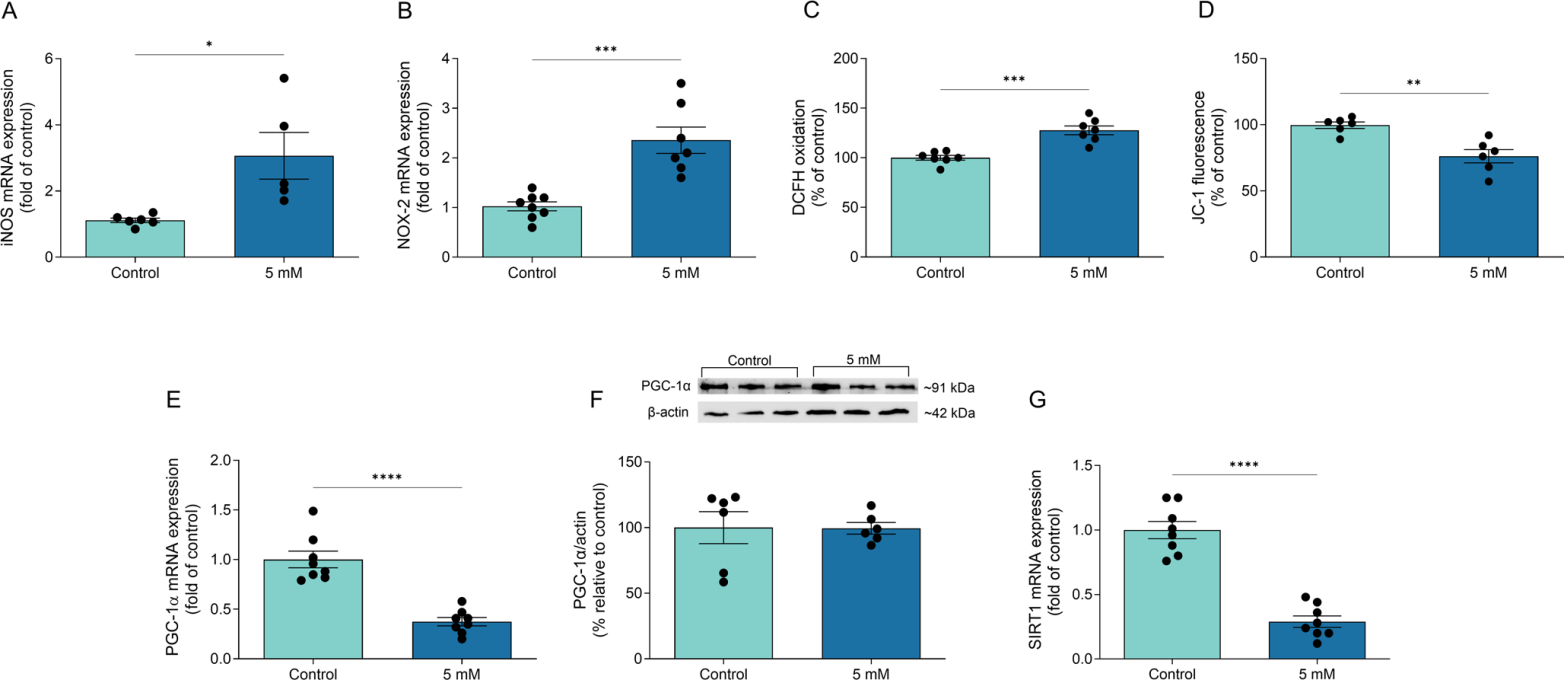
Effects of MMA on oxidative stress and mechanisms associated with inflammation and redox homeostasis. C6 astroglial cells were exposed to 5 mM MMA for 48 h and the mRNA expression of iNOS (**A**), NOX-2 (**B**), PGC-1α (**E**), and SIRT1 (**G**); the DCFH oxidation (**C**); the JC-1 fluorescence (**D**); the protein content of PGC-1α (**F**) were evaluated. Results are presented as mean ± standard deviation (S.D.) of at least five independent experiments performed in duplicate. Differences between groups were statistically analyzed using Student’s t-test for independent samples. Values of *p* < 0.05 were considered statistically significant (** p < 0.01, *** p < 0.001, and **** p < 0.0001)

MMA increased the mRNA expression of enzymes associated with neuroinflammation and redox homeostasis, including iNOS [t(9) = 3.048, *p* = 0.0138; Fig. 3A] and NOX-2 [t(13) = 5.039, *p* = 0.0002; Fig. 3B]. These enzymes are closely associated with oxidative and nitrosative stress. Consistently, MMA increased ROS production [t(12) = 5.534, *p* = 0.0001; Fig. 3C] and reduced mitochondrial membrane potential (ΔΨm) [t(10) = 4.198, *p* = 0.0018; Fig. 3D]. In parallel, the regulator of mitochondrial function and biogenesis, PGC-1α, exhibited a significant decrease in mRNA expression [t(14) = 6.651, *p* < 0.0001; Fig. 3E], whereas no significant changes were observed in protein levels (Fig. 3F). In addition, SIRT1, which plays a significant role in the regulation of inflammation, redox homeostasis, and metabolic processes, was significantly altered by MMA[t(14) = 8.880, *p* < 0.0001; Fig. 3G].

### MMA Modulated Glutamate Metabolism and GDNF Expression

**Fig. 4:**
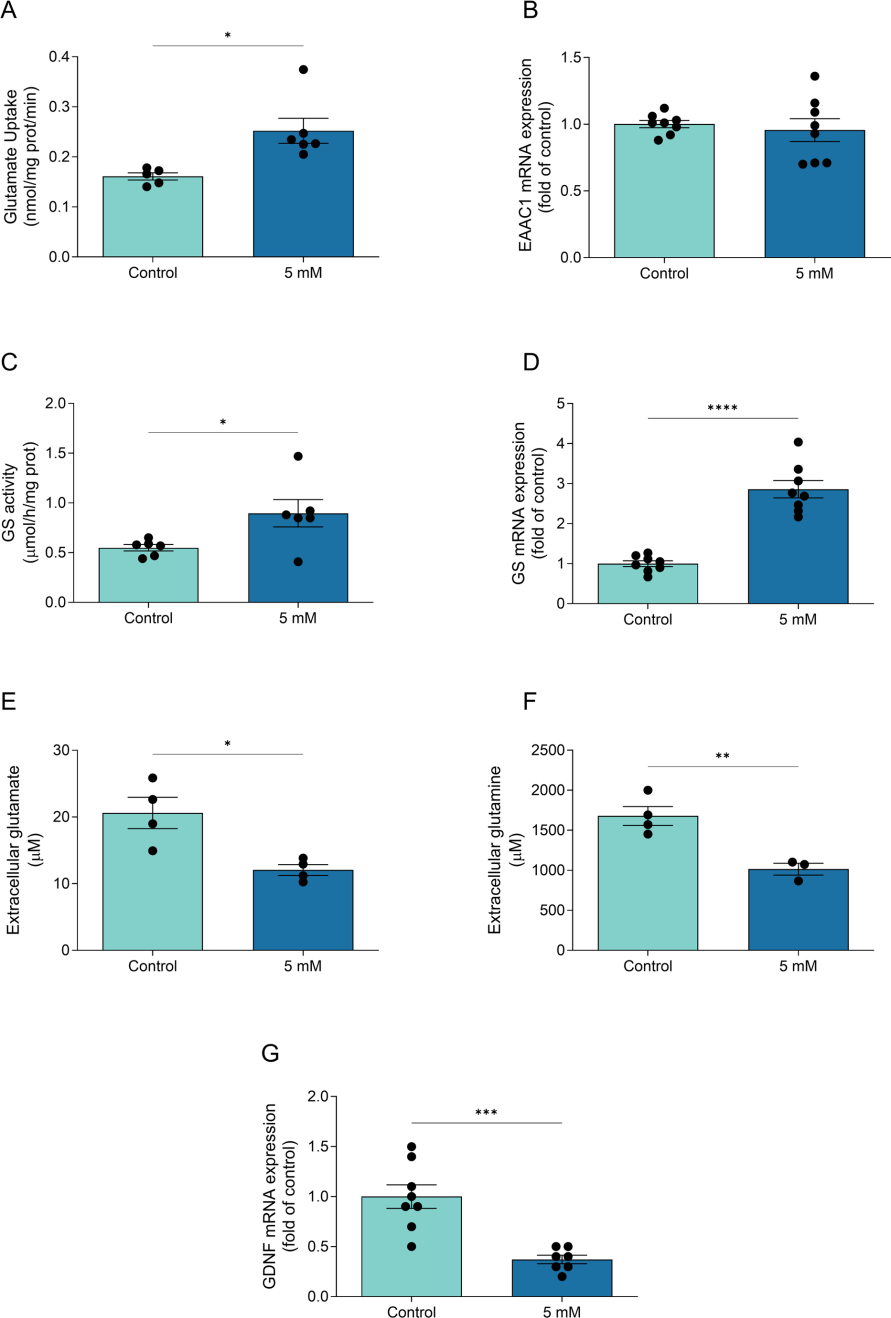
MMA alters glutamate metabolism and GDNF trophic factors. C6 astroglial cells were exposed to 5 mM MMA for 48 h and gene expression and metabolic parameters were assessed. **A** glutamate uptake, (**B**) and mRNA expression of EAAC1, (**C**) GS activity, (**D**) mRNA expression of GS, (**E**) extracellular levels of glutamate, (**F**) extracellular levels of glutamine, (**G**) mRNA expression of GDNF. Results are presented as mean ± standard deviation (S.D.) of at least four independent experiments performed in duplicate. Differences between groups were statistically analyzed using Student’s t-test for independent samples. Values of *p* < 0.05 were considered statistically significant (* p < 0.05, ** p < 0.01, *** p < 0.001, and **** p < 0.0001)

We then analyzed glial parameters related to astroglial functionality. In this regard, the incubation of astroglial cells with MMA resulted in increased glutamate uptake [t(9) = 3.195, *p* = 0.0109; Fig. 4A], with no effect in the mRNA levels of EAAC1 [t(14) = 0.5012, *p* = 0.624; Fig. 4B], the glutamate transporter endogenously expressed in C6 cells (Dowd and Robinson 1996). Once taken up by astroglial cells, glutamate is converted into glutamine by the enzyme GS, whose activity and mRNA expression increased by MMA exposure [GS activity: t(10) = 2.447, *p* = 0.0344; Fig. 4C; GS expression: t(14) = 8.144, *p* < 0.0001; Fig. 4D]. We also evaluated the extracellular levels of glutamate and glutamine, which were both decreased after MMA treatment [glutamate: t(6) = 3.431, *p* = 0.0104; Fig. 4E; glutamine: t(5) = 4.356, *p* = 0.0073; Fig. 4F]. Note that the DMEM used is supplemented with glutamine at a concentration of 4 mM. After 48 h of incubation, we observed mean extracellular glutamine concentrations of 1.68 mM and 1.01 mM in control and MMA-exposed cells, respectively, indicating substantial glutamine consumption by C6 cells under basal conditions, which appears to be further increased by MMA. Finally, MMA downregulated the mRNA expression of GDNF [t(13) = 4.738, *p* = 0.0004; Fig. 4G], a neurotrophic factor associated with glutamate metabolism.

## Discussion

Methylmalonic acidemia is a severe intoxicating metabolic disorder characterized by predominantly tissue accumulation of MMA, which has been strongly associated with neurological dysfunction (Gabbi et al. 2017; Chen et al. 2023). However, as patients experience progressive neurological damage resulting from chronic exposure to the metabolite, there is a need to elucidate the persistent deleterious cellular mechanisms that remain poorly understood. In a previous study from our group (de Souza Almeida et al. 2022), MMA (24 h of exposure) induced a redox imbalance associated with reduced antioxidant defenses, accompanied by increased Nrf2 expression, likely reflecting an adaptive response to restore redox homeostasis. In this study, we exposed C6 astroglial cells for 48 h to assess whether the previously observed short-lived effects are persistent and, importantly, whether novel mechanisms not yet investigated, involving metabolic regulators, glutamatergic pathways, and trophic factors, emerge by prolonged periods. We observed that both Nrf2 expression and its nuclear content are decreased, together with increased NOX-2 expression and reactive species production (DCFH oxidation), indicating a disruption of redox regulation under prolonged metabolic stress. Regarding inflammatory parameters, the effects at 24 h and 48 h are largely consistent, supporting a sustained inflammatory response. Therefore, we propose that this experimental approach reflects a more realistic approximation of the clinical chronic condition and reinforce the role of astrocytes as critical targets in MMA-induced toxicity. Considering that the C6 lineage has an average lifespan in culture of approximately 48–72 h, the exposure period adopted can be interpreted as a prolonged treatment within the temporal window of this cell lineage. Therefore, our focus was the assessment of inflammatory responses and glutamatergic parameters in a prolonged in vitro model to gain insights into the mechanisms underlying early neurodegenerative changes.

Astrocytes can offer protection against potential CNS damage in various disorders; however, they can also contribute to neurodegeneration, because they might exacerbate the inflammatory response. IL-1β is a potent pro-inflammatory cytokine with a crucial role in inflammation, which is a feature of several inborn errors of metabolism (IEM). Here, we observed a significant increase in both the expression and release of IL-1β, in agreement with our previous study using a shorter incubation period (24 h) with MMA (de Souza Almeida et al. 2022). Notably, IL1R1 gene expression was also elevated. The persistent activation of these genes is closely associated with the pathogenesis and progression of neurodegeneration, as it drives significant neuroinflammatory responses in the CNS. IL1R1 plays a central role in inducing other pro-inflammatory mediators, such as IL-6 (Basu et al. 2002; Luís et al. 2022). This is in accordance with our results showing a significant increase in IL-6 expression following MMA exposure. Although IL-6 may exert both pro- and anti-inflammatory effects, the concomitant rise in other cytokines supports a predominantly pro-inflammatory role in this context. Moreover, we observed a decrease in IL-10 mRNA levels, a cytokine that is known for its immunoregulatory properties and its role in mitigating inflammation-induced neurodegeneration (Porro et al. 2020). This finding may indicate a suppressive mechanism by which MMA sustains a chronic pro-inflammatory state, potentially impairing astroglial support functions through cellular and molecular damage. Interestingly, while inflammatory responses typically result in elevated TNF-α levels, our data show no significant changes in either TNF-α expression or in its receptor TNFR1 at 48 h of MMA exposure. This likely reflects time-dependent regulatory mechanisms rather than a resolution of inflammation, as evidenced by the persistence of other inflammatory markers. This pattern is consistent with previous reports showing that TNF-α production is tightly regulated, including through mRNA destabilization and feedback inhibitory loops (Frasca et al. 2012; Wallach 2016; Rothschild et al. 2018). Moreover, prolonged stimulation may induce a state of cellular desensitization, leading to reduced cytokine expression over time.

Key enzymes involved in the inflammatory processes include iNOS and COX-2. Consistent with this, we found that the expression of COX-2 mRNA was notably elevated in C6 astroglial cells under MMA treatment. This is a rate-limiting enzyme in the conversion of arachidonic acid to prostaglandins, and its increased expression levels can indicate its critical role in the prolonged neuroinflammatory processes (Hirst et al. 1999). MMA increased the mRNA levels of iNOS, reinforcing our previous findings. In addition, iNOS is particularly relevant in methylmalonic acidemia, because it is associated with the production of reactive nitrogen species as previously demonstrated (Ribeiro et al. 2009).

In the CNS, the inflammatory response triggered by tissue injury or neurodegeneration involves a complex interplay of signaling pathways with critical regulatory points. Among them, the transcription factor NFκB stands out as particularly significant, as it is implicated in the pathology of neuroinflammation, primarily through its regulation of pro-inflammatory cytokine production (Yu et al. 2020). Our findings indicate that MMA persistently increases NFκB activity, as evidenced by enhanced p65 NFκB gene expression and increased nuclear localization of p65 NFκB, indicative of nuclear translocation rather than changes in total protein abundance. These results corroborate previous findings from our group showing increased nuclear p65 NFκB levels in astroglial cells following MMA exposure (de Souza Almeida et al. 2022) and are consistent with recent evidence highlighting nuclear NFκB activation as a key feature of reactive astrocyte responses (Hein et al. 2026). This sustained upregulation may contribute to the induction of pro-inflammatory cytokines IL-1β and IL-6, while also being associated with downregulation of the anti-inflammatory cytokine IL-10. Notably, increased p65 NFκB activity was accompanied by enhanced p38 MAPK mRNA expression. Although our data do not provide direct evidence of p38 MAPK activation, these findings may suggest the involvement of this pathway in the inflammatory response induced by MMA, since p38 MAPK is involved in cellular responses to environmental stress and inflammatory stimuli (Cuenda and Rousseau 2007; Wang et al. 2024). Moreover, previous studies have shown that p38 MAPK signaling can modulate NFκB activity, contributing to the amplification of inflammatory responses and progression of brain injury (Guo et al. 2024).

Previous studies performed in patients with methylmalonic acidemia revealed increased levels of C reactive protein (CRP), IL-6, IL-8, TNF-α, IL-10, neural cell adhesion molecule 1 (NCAM-1), and cathepsin-D in plasma of these patients, indicating systemic inflammation (Dos Reis et al. 2024). Our study not only reinforces the involvement of inflammation in the pathophysiology of methylmalonic acidemia but also reveals novel inflammatory pathways activated by MMA in astroglial cells that lead to neuroinflammation. Specifically, we found that MMA induces alternative inflammatory responses through increased expression of NOX-2, an enzyme implicated in superoxide production, highlighting a crosstalk between oxidative stress and the inflammatory cascade (Ardizzone et al. 2024). Furthermore, changes in redox balance correlate with prolonged reduction of PGC-1α mRNA expression, a critical regulator of mitochondrial function, whereas its protein content remained unchanged at the time point analyzed. Reduced PGC-1α transcription may contribute to inflammatory and metabolic disturbances. In addition, lower PGC-1α transcript levels may be associated with decreased expression of mitochondrial antioxidant genes, which has been linked to NFκB signaling activation (Shelbayeh et al. 2023). This discrepancy between transcript and protein levels may be explained by post-transcriptional regulatory mechanisms and the temporal dynamics of protein turnover during metabolic stress, which can preserve PGC-1α protein levels despite reduced transcription (Coppi et al. 2021; Shelbayeh et al. 2023). On the other hand, MMA also decreases SIRT1 gene expression, reinforcing that this organic acid significantly changes metabolic and stress responses, including SIRT1-mediated regulation of inflammation (Yang et al. 2022).

Inflammatory responses and glutamate uptake disruption are interconnected processes that often arise as consequences of brain injury, potentially exacerbating glial damage in the CNS by the regulation of different signaling pathways (Haroon et al. 2017). Notably, exposure to MMA has been shown to increase glutamate uptake, aligning with previous studies that reported enhanced glutamate uptake in astrocytes and the cerebral cortex following exposure to other organic acids (Junqueira et al. 2003; Frizzo et al. 2004). This increase may reflect a pathophysiological response of astrocytes to MMA. However, this change in glutamate uptake does not correlate with alterations in EAAC1 mRNA expression, the endogenous glutamate transporter of C6 astroglial cells, suggesting that MMA directly affects glutamate transport activity rather than altering transporter expression. EAAC1 function can be regulated at levels beyond gene and protein expression, including post-translational mechanisms and changes in transporter activity or localization (Dowd and Robinson 1996), which may explain the lack of changes in its expression despite the observed increase in glutamate uptake.

Furthermore, we observed an increase in GS gene expression and activity following MMA exposure. Despite this, extracellular glutamine levels were reduced, suggesting their possible utilization as alternative energy substrates (Mahmoud et al. 2019; Andersen and Schousboe 2023). It is important to note that MMA is known to impair tricarboxylic acid cycle function and disrupt anaplerotic flux (Mirandola et al. 2008; Kielar et al. 2009; Wongkittichote et al. 2019), which may lead to increased glutamate and glutamine utilization not only for energy production but also as an anaplerotic substrates. In this context, the observed increase in GS (which uses glutamate and ammonia as substrates) may represent a compensatory response to elevated ammonia levels generated by enhanced glutamate and glutamine catabolism via glutaminase and glutamate dehydrogenase. Although direct evidence on the effects of MMA on glutamine metabolism is limited, previous studies report hyperammonemia as a common feature in patients with methylmalonic acidemia (Wajner 2019), along with broader disruptions in amino acid metabolism (Rajabi et al. 2019). Therefore, it is plausible that increased GS activity reflects a compensatory response to ammonia accumulation, while the reduction in extracellular glutamine results from its increased intracellular utilization.

Trophic factors such as GDNF can modulate glial cells and may offer neuroprotection by regulating both inflammatory and metabolic functions (Singh et al. 2023). In particular, GDNF has been shown to influence glutamate uptake and metabolism in astrocytes, thereby contributing to the maintenance of excitatory neurotransmitter homeostasis under stress conditions (Farrand et al. 2015). In our model, long-term MMA exposure led to a reduction in GDNF expression, which may impair glutamatergic regulation. This effect may also be associated with late development or dysfunction of different neuronal subtypes (Duarte Azevedo et al. 2020; de Souza Almeida et al. 2022), highlighting the potential role of GDNF in the progression of neurological deterioration observed in patients with this IEM.

Biological systems are inherently dynamic, and the temporal sequence of molecular events is critical for understanding how cells integrate signals, adapt to environmental changes, and transition between functional states. In this context, distinguishing between acute and prolonged effects is essential, as these can provide important insights into developmental processes, disease progression, and the extent to which transient molecular signals drive long-term cellular outcomes. The present study has limitations that should be considered. Although in vitro experimental models do not fully recapitulate the complexity of the brain, they represent well-established systems for investigating cell signaling pathways, gene expression, and intrinsic glial functions, particularly under conditions of metabolic stress. In addition, SIRT1 and p38 MAPK were assessed only at the mRNA level, and the absence of protein phosphorylation analyses for p38 MAPK limits conclusions regarding the functional activation status of these signaling pathways. Nonetheless, further studies using in vivo approaches, as well as experimental designs addressing time- and dose-dependent responses, are required to validate and expand upon the effects of MMA described here.

Considering these aspects, the present findings provide important insights into the cellular consequences of sustained MMA exposure, particularly in astroglial cells. Understanding the influence of MMA on astroglial cells is essential to elucidate the cellular and molecular mechanisms underlying the effects of this metabolite on the CNS. In this study, we demonstrated that prolonged in vitro exposure to this toxic metabolite directly affects key astroglial functions, including the inflammatory response, trophic support, and glutamate metabolism. Furthermore, we observed alterations in the expression of critical signaling pathways, such as Nr2/HO-1, NFκB, p38 MAPK, SIRT1, NOX-2, and PGC-1α. These changes may significantly contribute to understanding the pathomechanisms involved in the pathogenesis of methylmalonic acidemia and, consequently, how IEM-induced persistent gliotoxicity impacts patients throughout life.

## Supplementary Information

Below is the link to the electronic supplementary material.

Supplementary figure 1 (PNG 80.6 KB)

High Resolution Image (TIF 834 KB)

Supplementary figure 2 (PNG 127 KB)

High Resolution Image (TIF 1.17 MB)

Supplementary Material 3

## Data Availability

The datasets used and/or analyzed during the current study are available from the corresponding author upon reasonable request.
