# Supplementary figures and images for: Prolonged In Vitro Exposure to Methylmalonic Acid Induces Inflammation, Glutamate Metabolism Disruption, and Alters Functional Gene Expression in C6 Astroglial Cells
Source: Neurotox Res. 2026 Jun 23;44(4):28. doi: 10.1007/s12640-026-00806-1 (PMC13290939; doi:10.1007/s12640-026-00806-1)

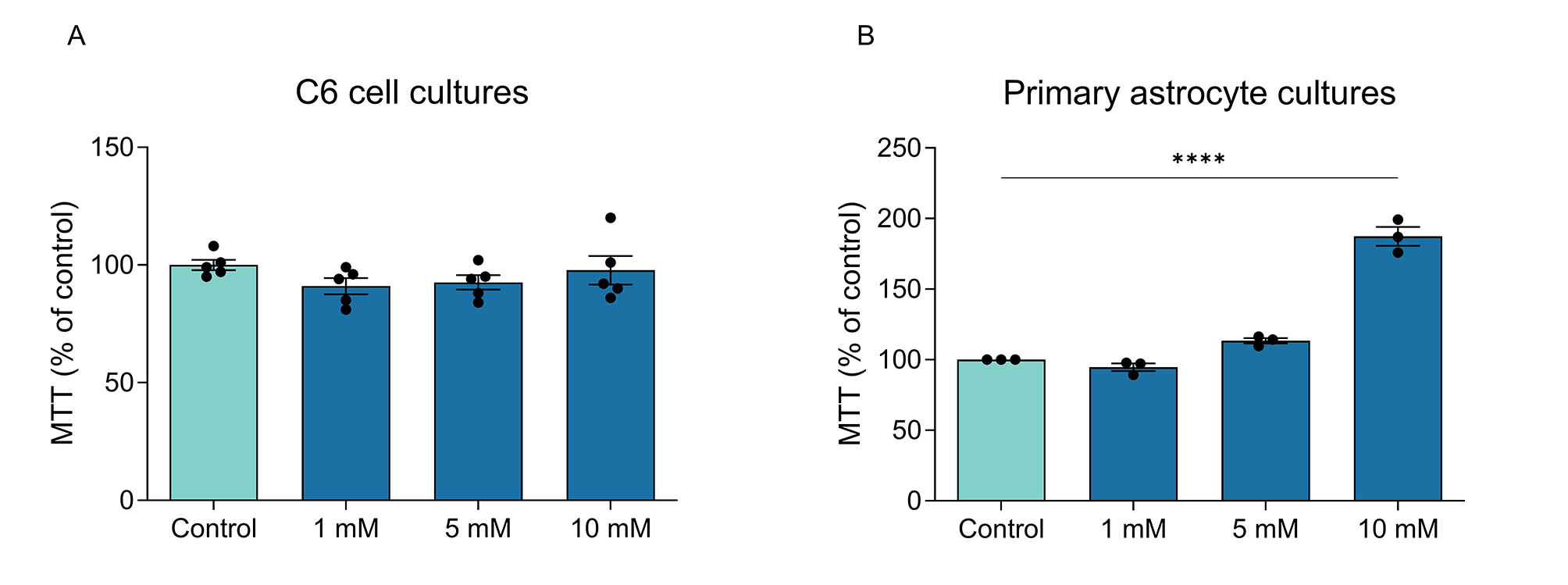

Supplement: Supplementary file 1 — Supplementary figure 1 (PNG 80.6 KB) [file 12640_2026_806_Fig5_ESM.png]

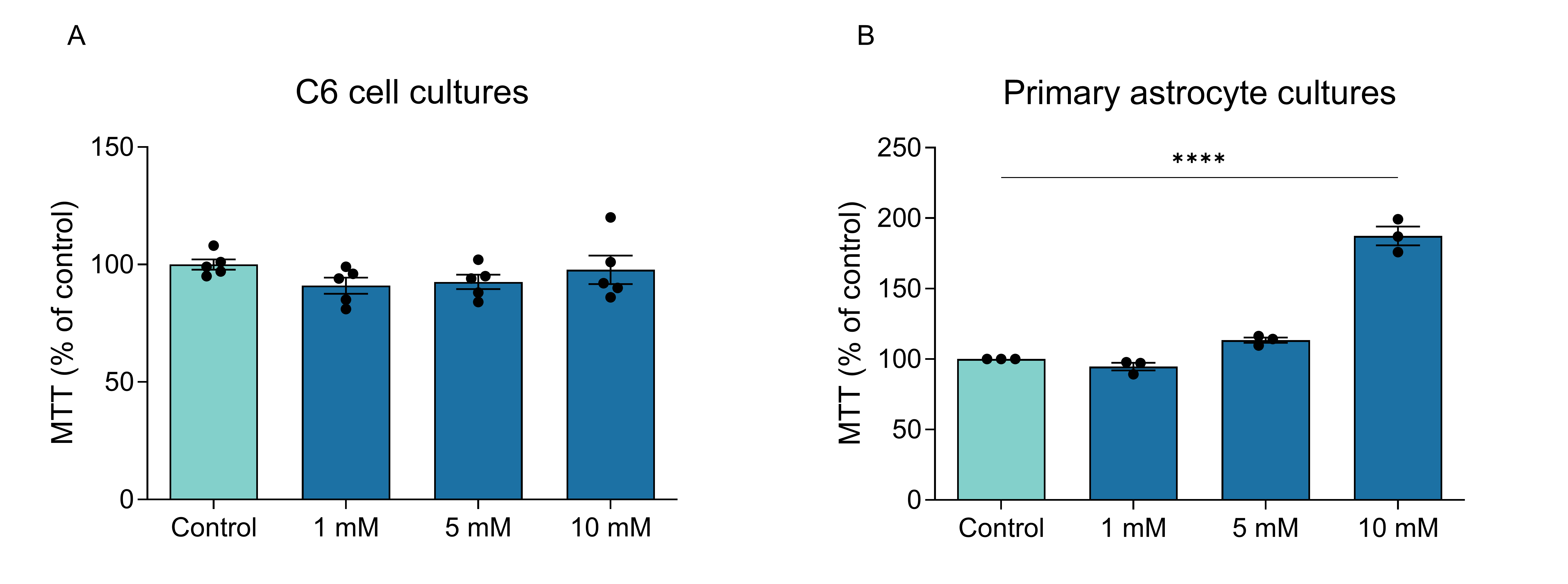

Supplement: Supplementary file 2 — High Resolution Image (TIF 834 KB) [file 12640_2026_806_MOESM1_ESM.tif]

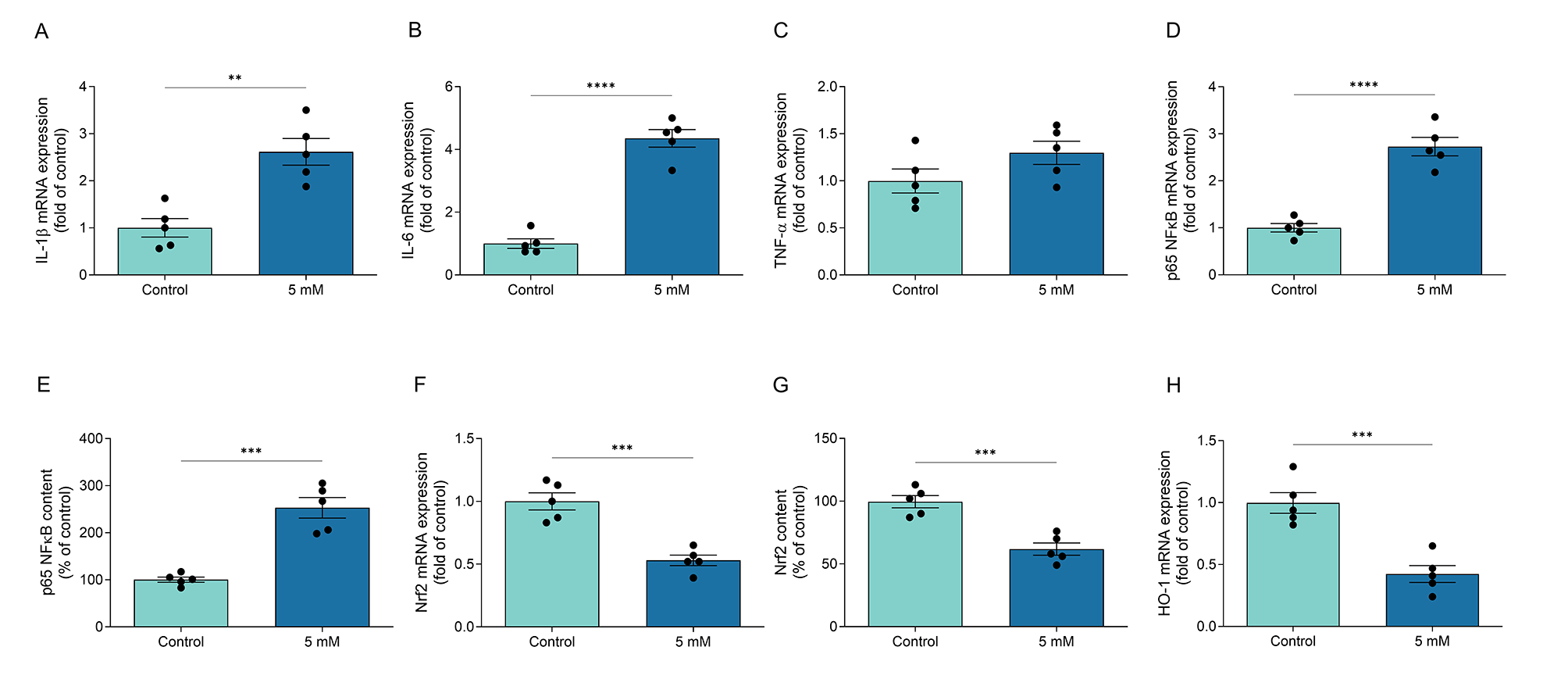

Supplement: Supplementary file 3 — Supplementary figure 2 (PNG 127 KB) [file 12640_2026_806_Fig6_ESM.png]

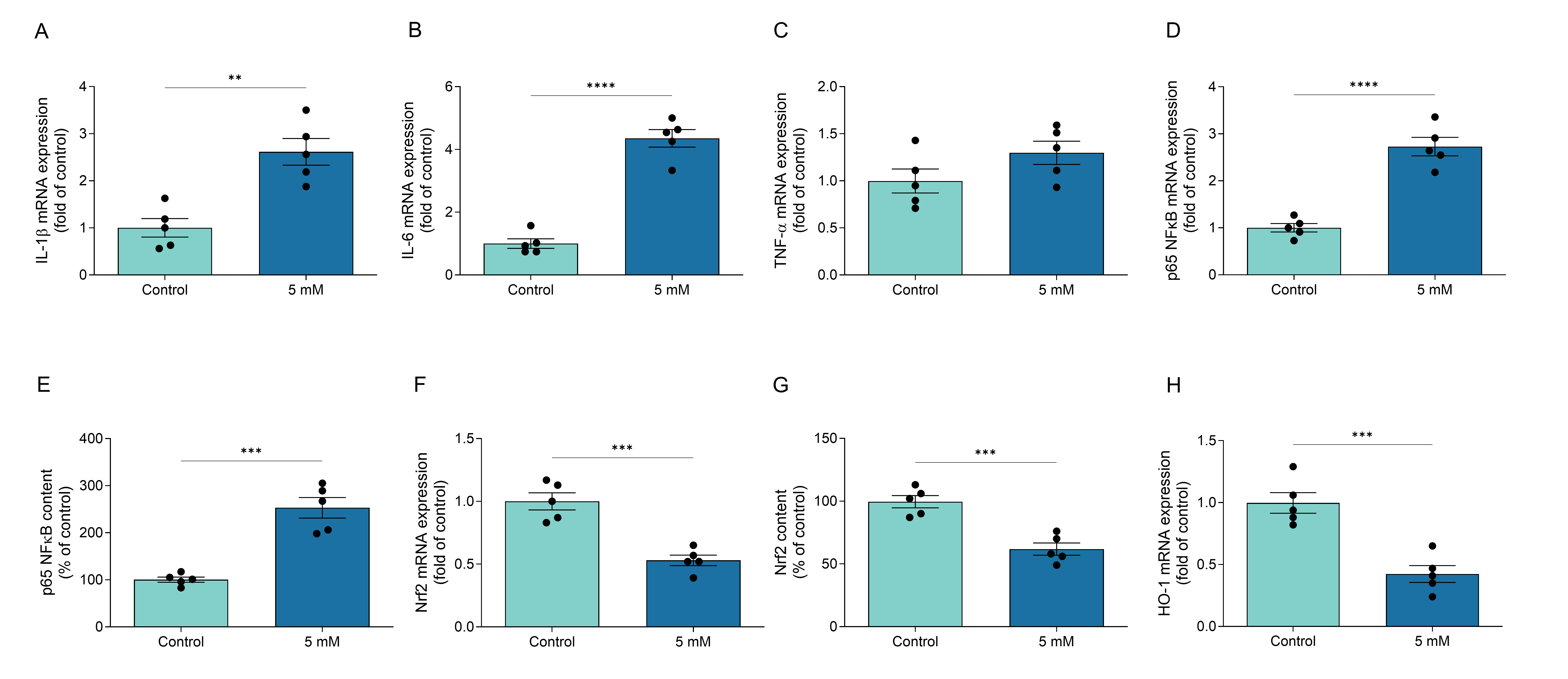

Supplement: Supplementary file 4 — High Resolution Image (TIF 1.17 MB) [file 12640_2026_806_MOESM2_ESM.tif]

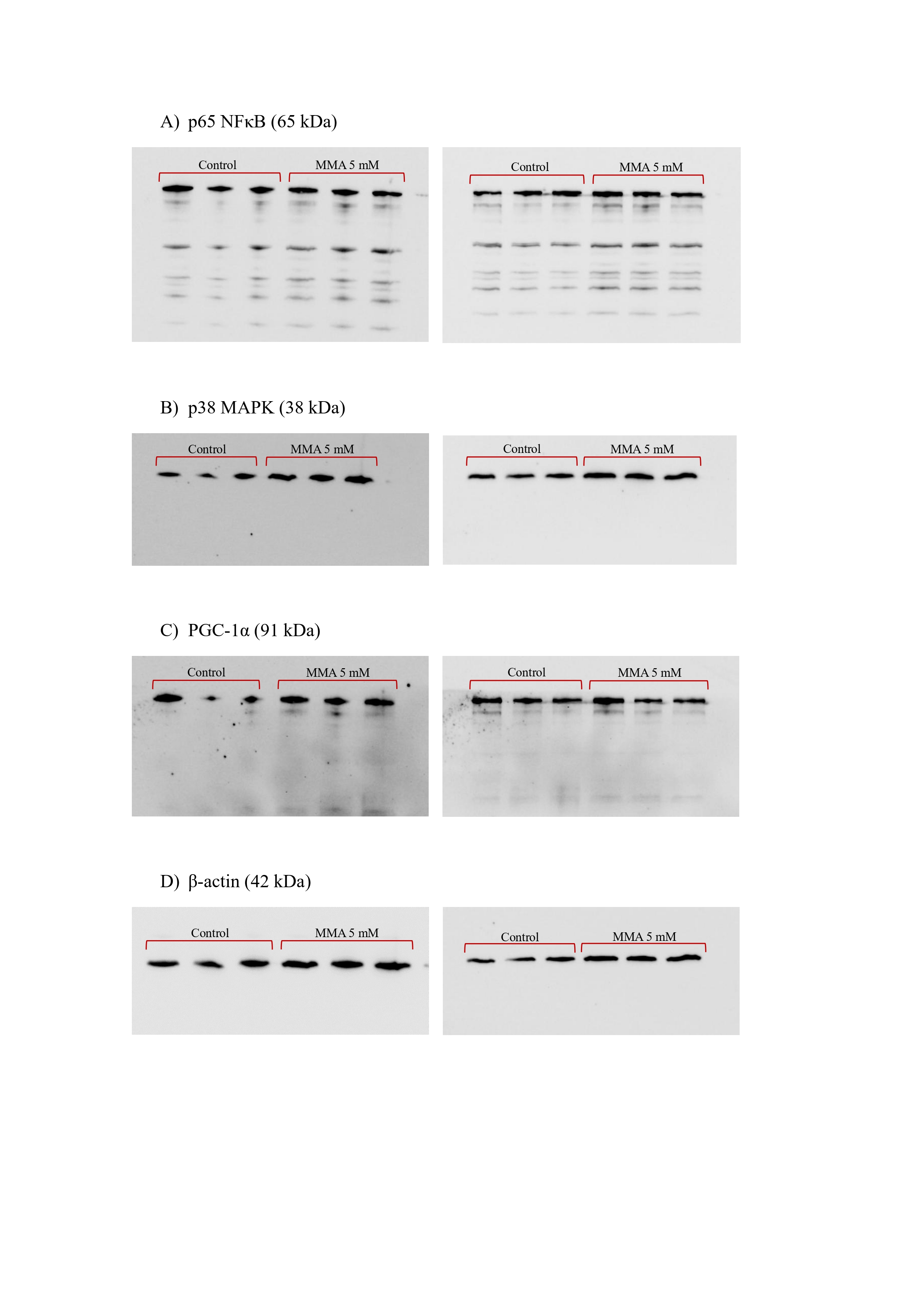

Supplement: Supplementary file 5 — Supplementary Material 3 [file 12640_2026_806_MOESM3_ESM.jpg]
